# Identification of a stable complex between a [NiFe]‐hydrogenase catalytic subunit and its maturation protease

**DOI:** 10.1002/1873-3468.12540

**Published:** 2017-01-11

**Authors:** Marta Albareda, Grant Buchanan, Frank Sargent

**Affiliations:** ^1^ School of Life SciencesUniversity of DundeeUK; ^2^ Present address: Departamento de Biotecnología‐Biología VegetalEscuela Técnica Superior de Ingeniería Agronómica, Alimentaria y de BiosistemasCentro de Biotecnología y Genómica de Plantas (C.B.G.P.)Universidad Politécnica de MadridCampus de Montegancedo, Carretera M40‐km 3828223Pozuelo de AlarcónMadridSpain

**Keywords:** anaerobic respiration, bacterial hydrogen metabolism, metalloenzyme biosynthesis, [NiFe]‐hydrogenase, protein–protein interactions, *Salmonella enterica*

## Abstract

*Salmonella enterica* serovar Typhimurium has the ability to use molecular hydrogen as a respiratory electron donor. This is facilitated by three [NiFe]‐hydrogenases termed Hyd‐1, Hyd‐2, and Hyd‐5. Hyd‐1 and Hyd‐5 are homologous oxygen‐tolerant [NiFe]‐hydrogenases. A critical step in the biosynthesis of a [NiFe]‐hydrogenase is the proteolytic processing of the catalytic subunit. In this work, the role of the maturation protease encoded within the Hyd‐5 operon, HydD, was found to be partially complemented by the maturation protease encoded in the Hyd‐1 operon, HyaD. In addition, both maturation proteases were shown to form stable complexes, *in vivo* and *in vitro*, with the catalytic subunit of Hyd‐5. The protein–protein interactions were not detectable in a strain that could not make the enzyme metallocofactor.

## Abbreviations

**HA**, hemagglutinin

**IMAC**, immobilized metal ion affinity chromatography

**RBS**, ribosome‐binding site

The respiration of H_2_ is important during the infection process followed by *Salmonella enterica* serovar Typhimurium 1, 2, 3. The *S. enterica* genome encodes four [NiFe]‐hydrogenases, termed Hyd‐1, Hyd‐2, Hyd‐3, and Hyd‐5. Hyd‐1 and Hyd‐2 are known or predicted to be involved in respiratory H_2_ oxidation 4, 5. Both are synthesized strictly under anaerobic conditions and *S. enterica* Hyd‐1 is closely related in terms of protein sequence and genetic structure to *Escherichia coli* Hyd‐1, which is an oxygen‐tolerant enzyme 6. The *S. enterica* Hyd‐5 isoenzyme shares structural similarities with Hyd‐1, but is synthesized under aerobic conditions 7, 8 and mouse model studies have shown that *S*.* enterica* Hyd‐5 is expressed during infection 3, 9.

The Hyd‐5 isoenzyme is encoded by *hydABCDEFGHI* (STM1539‐STM1531) 7 and *hydABC* encodes the complete enzyme. The core catalytic subunits comprise a large subunit (~ 60 kDa, HydB), that carries the catalytic cofactor [NiFe(CN^−^)_2_CO], and a small subunit (~ 35 kDa, HydA) containing three Fe‐S clusters 10.

Biosynthesis of the NiFe(CN)_2_CO active site cofactor, and its insertion into the precursor of the large subunit, requires the action of specialist accessory proteins 11. Once the [NiFe] cofactor is in place within the large subunit it is proteolytically processed at its C terminus by another accessory protein, a specific endopeptidase, the action of which renders the cofactor loading pathway irreversible 12. Proteolytic processing of [NiFe]‐hydrogenases is critical and without it such enzymes remain completely inactive. Genetic and biochemical studies have suggested that each individual hydrogenase isoenzyme is processed by its own dedicated endopeptidase 13, 14, 15. These accessory proteins typically remove an approximately 15 amino acid residue peptide (‘assembly peptide’) from the C terminus of the large subunit following a conserved histidine residue within the consensus motif DPCXXCXXH, the cysteines of which provide two of the ligands needed for co‐ordination of the [NiFe] cofactor.

The crystal structures of processing proteases revealed metal‐binding enzymes. *E. coli* HybD, for example, contains cadmium ions in the structure 16, and *E. coli* HycI has a calcium ion binding site 17, 18. Proteolytic cleavage of the large subunit by the endopeptidase has been considered ‘nickel dependent’ in so far as the [NiFe] cofactor must be loaded into the large subunit before proteolysis 19. Moreover, because purified proteases do not contain metal, and metal binding has been considered a crystallographic artifact 11, 16, 17, it has been hypothesized that the metal‐binding motif of the endopeptidases is used to recognize the presence of the complete [NiFe] cofactor within the large subunit precursor 11.

In this work, the *S. enterica* Hyd‐5 system has been employed to further understand the relationship between a [NiFe]‐hydrogenase large subunit and its cognate maturation endopeptidase. For Hyd‐5, the maturation protease was predicted to be encoded by the *hydD* gene. Here, deletion of *hydD* is shown to hamper Hyd‐5 biosynthesis, but HydD function can be partially rescued by the HyaD protein encoded within the operon for Hyd‐1. Furthermore, new evidence is provided that HydD, and HyaD, can form unexpectedly stable complexes with the large subunit of Hyd‐5. Surprisingly, genetic experiments suggest the interactions do not require the presence of the C‐terminal extension on HydB. Indeed, stable HydB‐HydD and HydB‐HyaD complexes can be isolated where the C‐terminal extension of HydB remains completely unprocessed. These data suggest more elaborate roles for these important accessory proteins beyond transient proteolysis.

## Materials and methods

### Bacterial growth and plasmids

Strains constructed in this work are listed in Table S1. *S. enterica* strains were grown in ‘low salt’ LB (5 g·L^−1^ NaCl) media while *E*. *coli* strains were cultured in standard LB (10 g·L^−1^ NaCl) medium. Final antibiotic concentrations were used as follows: ampicillin, 125 μg·mL^−1^; chloramphenicol, 12.5 μg·mL^−1^ (for *S. enterica*) or 25 μg·mL^−1^ (for *E*.* coli*).

Plasmids constructed and studied in this work are listed in Table S2. For construction of plasmids pBADHyaD and pBADHydD, the *hyaD* and *hydD* genes were amplified by PCR and cloned in vector pBAD24 20 using *Eco*RI‐*Sal*I. Plasmids for bacterial two‐hybrid analysis were constructed using the pUT18 and pT25 vectors 21. The *hyaD* and *hydD* genes were PCR amplified, minus stop codons, and cloned into pUT18 using *Bam*HI‐*Eco*RI sites. The *hydB* gene was PCR‐amplified and cloned into pT25 using *Pst*I‐*Bam*HI sites. The first truncated form, HydB_T1_, was amplified and cloned into pT25 vector using *Bam*HI‐*Sma*I. The second truncated form, HydB_T2_, was cloned as a *Bam*HI and *Kpn*I fragment into the pT25 vector.

For copurification experiments, vectors were designed to allow HydB to be overproduced along with either HyaD or HydD His‐tagged variants. The *hydB* gene, including the initiation codon but with the native UGA stop codon replaced by two consecutive UAA stop codons to prevent read‐through, was amplified using primers described in Table S3. The forward primer included an artificial ribosome‐binding site (RBS) and six‐base spacer to the initiation codon. The PCR product was digested with *Eco*RI and *Sph*I and cloned into pQE80 resulting in pQE80‐HydB. Then, *hyaD* or *hydD* were PCR‐amplified using forward primers that included an artificial RBS and six‐base spacer to the initiation codon. The reverse primers included the sequence for the hexahistidine affinity tag. PCR products were separately cloned into the pQE80‐HydB vector using *Hin*dIII‐*Pst*I sites, resulting in pQE80‐HydB‐HyaD_HIS_ or pQE80‐HydB‐HydD_HIS_ vectors.

### Mutant strain construction

Plasmids constructed in this work are listed in Table S2. Oligonucleotides used as primers are listed in Table S3. In‐frame deletions of chromosomal *hypD*,* hydD,* and *hyaD* genes were constructed using pMAK705 22. DNA covering approximately 500 bp upstream of the gene to be deleted, including the translation start site, was PCR amplified using genomic DNA from the LT2a strain as a template and cloned into pBluescript‐II KS^+^ using *Bam*HI‐*Eco*RI sites. Next, DNA covering 500 bp downstream of the gene, including the terminator site, was amplified and cloned into the new vector using *Eco*RI‐*Hin*dIII sites. The deletion alleles were moved into the pMAK705 vector as *Bam*HI/*Hin*dIII fragments and onto the chromosome of the *S. enterica* LB03 strain by homologous recombination 22.

The construction of LB03 derivative strains (Δ*hypD*, Δ*hyaD*, Δ*hydD,* and Δ*hyaD* Δ*hydD*) encoding a hemagglutinin (HA) tag at the N terminus of HydB was performed as follows. A first round of PCR reactions amplified DNA covering the up and downstream of the *hydB* gene using primers 1HA tag HydB _SacI_FW/2HA tag HydB _RV and 3HA tag HydB _FW/4HA tag HydB_XbaI_RV primers, respectively. The two PCR products were linked *via* their overlapping sequence coding for the HA tag (YPYDVPDYA) fused in frame in the corresponding site of *hydB* using primers 1HA tag HydB _SacI_FW/4HA tag HydB_XbaI_RV. PCR products were cloned in pBluescript‐II KS^+^ as *Sac*I‐*Xba*I fragments, moved to pMAK705 with *Sac*I/*Xba*I and the HA‐tag sequence incorporated into the chromosome by homologous recombination 22.

For *E. coli* MAE01 (MG1655 Δ*cyaA*::Apra^R^) 500 bp upstream of *cyaA* was amplified and cloned into pKS vector as an *Eco*RI‐*Xba*I fragment. Next, 500 bp downstream was amplified and cloned into the new vector using *Xba*I*‐Hin*dIII. Then, the deletion allele was subcloned into pMAK705 leading to yield pFGM1. An apramycin resistance cassette was amplified from pIJ773 23, digested with *Spe*I, and cloned into *Xba*I‐digested pFGM1 to give pFGM2 and the deletion was moved onto the MG1655 chromosome 22. The *E. coli* MG1655 (Δ*cyaA*::Apra^R^ Δ*hypF*::Kan^R^) strain (MAE02) was constructed by P1 transduction the Δ*hypF::Kan* allele from JW5433 24 into the MAE01.

### Hydrogenase activity assays

Hydrogenase activity was measured by H_2_‐dependent reduction of benzyl viologen 25. Starter cultures were grown aerobically in low salt LB at 37 °C. A 1 : 1000 dilution was made in 20 mL of fresh media and arabinose, when necessary, was added to 0.02% (w/v) final concentration. Cultures were incubated at 37 °C for 16 h anaerobically before washed cell pellets were suspended in 500 μL 50 mm Tris/HCl (pH 7.5). Protein concentration of cell suspensions was determined by a modified Lowry method 26 using the DC Protein Assay Kit (BioRad, Hercules, CA, USA).

### Rocket immunoelectrophoresis

Rocket immunoelectrophoresis was performed as described 8. Periplasmic fractions were obtained from 250 mL of bacterial cultures grown anaerobically in low‐salt LB at 37 °C for 16 h and prepared by a lysozyme/EDTA method 8.

### Bacterial two‐hybrid interaction assay

Bacterial two‐hybrid analysis was performed as described 27 except bacterial cultures were grown anaerobically at 30 °C for 16 h in static Hungate tubes filled with 10 mL of LB media. Protein–protein interactions were quantified by β‐galactosidase activity assays 28.

### Protein purification

Protein purification was carried using 5 L cultures of *E. coli* FTD147 (pQE80HydB‐HydD_HIS_) or FTD147 (pQE80HydB‐HyaD_HIS_) grown anaerobically at 37 °C in Duran bottles filled to the top with low salt LB media. Protein production was induced with 2 mm (final concentration) of isopropyl β‐d‐thiogalactopyranoside at the outset. After 16 h, cells were harvested and suspended in Buffer A (50 mm Tris/HCl, pH 7.5, 150 mm NaCl, and 75 mm imidazole) containing protease inhibitors (Complete‐mini, EDTA‐free; Roche, Basel, Switzerland), 50 μg·mL^−1^ of lysozyme and 10 μg·mL^−1^ of DNase I. Cells were broken using Emulsiflex‐C3 homogenizer and soluble fractions prepared by ultracentrifugation at 134 000 ***g*** at 4 °C. The soluble extract was loaded onto a 5 mL His Trap HP column (GE Healthcare, Little Chalfont, Buckinghamshire, UK) equilibrated with buffer A. Bound proteins were eluted with a 50‐mL linear gradient of the same buffer containing 500 mm imidazole. Fractions containing the His‐tagged proteins were pooled and concentrated in a Vivaspin 20 (Millipore Inc., Billerica, MA, USA) filtration device (10 kDa molecular weight cutoff).

### Protein analytical methods

Proteins were separated by standard SDS/PAGE 29 or Bis‐Tris gels 30. Proteins were visualized in‐gel using Instant Blue stain (Expedeon, San Diego, CA, USA) or, if necessary, were transferred to nitrocellulose 31. Western immunoblots were performed using an HRP‐conjugated anti‐hexaHis monoclonal (1 : 10 000 dilution; Abcam, Cambridge, UK) and an anti‐HA Epitope Tag Monoclonal (1 : 2000 dilution; Sigma‐Aldrich, St. Louis, MO, USA). Blots were developed using a Clarity Western ECL Substrate Kit (BioRad Laboratories) and data collected with the GeneGNOME camera system (Syngene, Cambridge, UK). For protein identification by tryptic peptide mass fingerprinting, protein samples of interest were resolved in 12% (w/v) acrylamide, stained with Instant Blue, and analyzed as a service by FingerPrints Proteomics Facility, University of Dundee.

## Results

### Evidence for overlapping roles of HydD and HyaD in the biosynthesis of Hyd‐5

Hyd‐5 is normally produced aerobically at very low levels 7, therefore the *S. enterica* LT2a strain was previously modified by the addition of a T5 promoter upstream of the *hyd* operon 7, and the transmembrane domain of the small subunit replaced by an affinity tag, generating LB03 8. This engineering was found to boost cellular hydrogenase activity in LB03 by five times in comparison to the basal activity recorded for LT2a (Table 1).

**Table 1:** Hydrogenase activity in *Salmonella enterica* strains in intact cells derived from anaerobic cultures

Strains	Genotype	Hydrogenase activity^a^
LT2a	Wild‐type	21 ± 6
LB03	P_T5_, *hydA* ^ΔTM‐His^	100 ± 7
MAS01	Δ*hypD*	No activity
MAS02	Δ*hydD*	66 ± 9
MAS03	Δ*hyaD*	92 ± 7
MAS04	Δ*hyaD* Δ*hydD*	19 ± 5
MAS02/pBADHydD	Δ*hydD* + *hydD*	95 ± 8
MAS03/pBADHyaD	Δ*hyaD* + *hyaD*	100 ± 15
MAS04/pBADHydD	Δ*hyaD* Δ*hydD* + *hydD*	73 ± 2
MAS04/pBADHyaD	Δ*hyaD* Δ*hydD* + *hyaD*	67 ± 1

^a^ Hydrogenase activities are expressed as percentages of the hydrogenase activity associated with LB03 parental strain. The absolute values (100%) of hydrogenase activity for this strain were 166 ± 12 nmol H_2_ oxidized·min^−1^·mg protein^−1^. Values are means of at least three independent assays ± SE.

The protein predicted to catalyze processing of Hyd‐5 is HydD (STM1536) and an in‐frame deletion in *hydD* was generated in LB03 to generate MAS02. Somewhat surprisingly, when the Δ*hydD* MAS02 strain was assayed, total hydrogenase activity levels were found to be 66% of that normally recorded for the parent strain (Table 1). Note that deletion of the *hypD* gene, which is essential for cofactor biosynthesis, completely abolished all hydrogenase activity (Table 1). The partial phenotype of Δ*hydD* could be complemented by *hydD* in trans (Table 1). These data indicate that HydD alone is not completely essential for Hyd‐5 activity. The large subunits of Hyd‐5 (HydB) and Hyd‐1 (HyaB, STM1787) share 67% overall sequence identity (75% overall similarity). The processing step of Hyd‐1 would be expected to be carried out by HyaD (STM1789), which itself shares 53% overall sequence identity with HydD. Thus, a double mutant (Δ*hyaD,* Δ*hydD*) was constructed (MAS04). The Δ*hyaD,* Δ*hydD* double mutant (MAS04) was found to be severely compromised in terms of Hyd‐5 activity and showed only basal activity levels, similar to that exhibited by the LT2a strain under these growth conditions (Table 1). The level of hydrogenase activity detected in the parental strain LT2a was attributed to the other hydrogenases expressed under these conditions (20% in relation to up‐regulated LB03 strain), indicating that the double mutant (MAS04) was not compromised in this regard, although the assembly of the other individual hydrogenases was not investigated here. The Δ*hyaD,* Δ*hydD* double mutant could be complemented to similar levels (67–73% of hydrogenase activity measured in parent strain) by introduction of either *hyaD* or *hydD* on a plasmid (Table 1).

### Engineering an epitope tag on the Hyd‐5 large subunit

**Figure 1:**
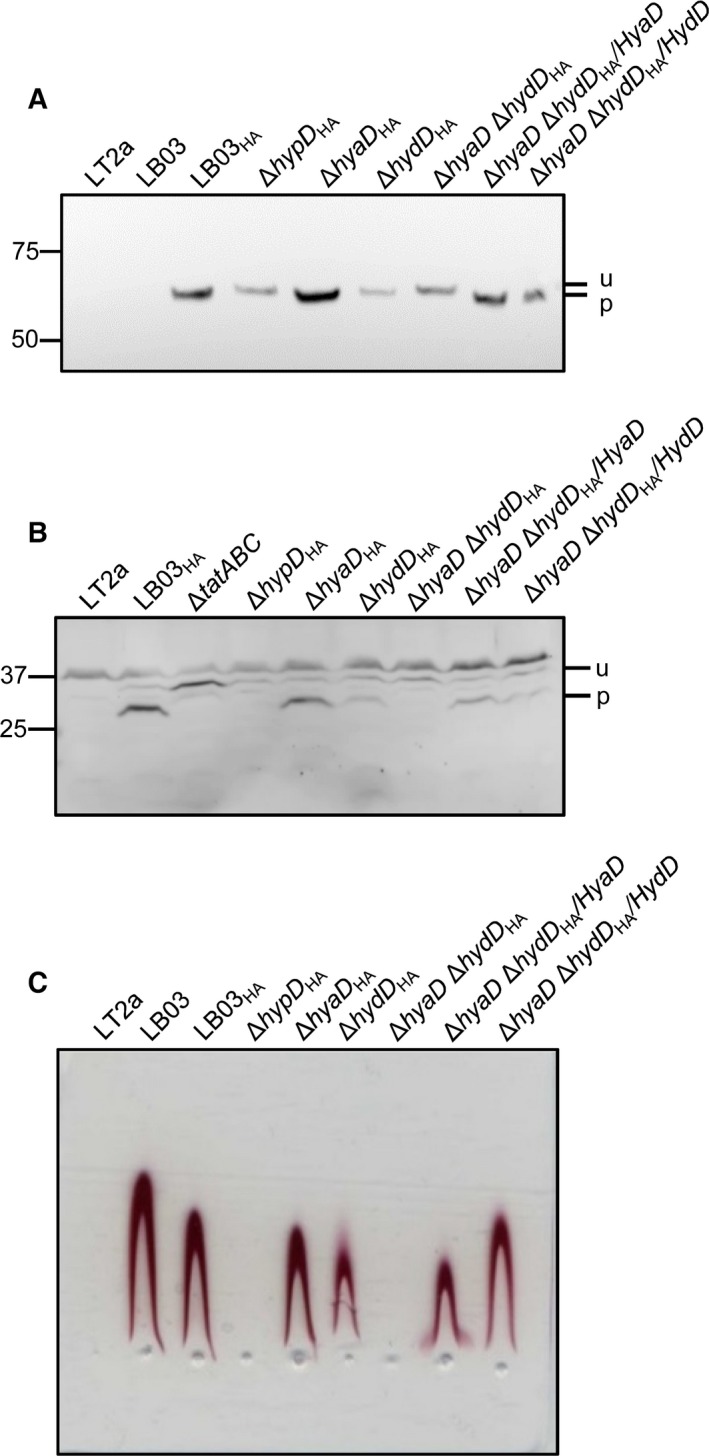
Cross‐talk between HydD and HyaD during the biosynthesis of Hyd‐5. *Salmonella enterica* strains LT2a (parental strain); LB03 (up‐regulated, His‐tagged Hyd‐5); LB03T (Δ*tatABC*); MAS05 producing HA‐tagged HydB (LB03_HA_); MAS06 (Δ*hypD*_HA_); MAS07 (Δ*hydD*_HA_); MAS08 (Δ*hyaD*_HA_); MAS09 (Δ*hyaD* Δ*hydD*_HA_); MAS09 complemented with pBAD‐HyaD (Δ*hyaD* Δ*hydD*_HA_/HyaD); and MAS09 complemented with pBADH‐ydD (Δ*hyaD* Δ*hydD*_HA_/HydD) were all grown anaerobically before being harvested, washed, and analyzed. (A) Immunodetection of the large subunit HydB_HA_. Whole‐cell protein samples were separated by SDS/PAGE (8% w/v acrylamide with a Bis‐Tris buffer system), blotted onto nitrocellulose, and challenged with an anti‐HA serum to detect the HydB_HA_ protein. Each lane was loaded with 60 μg of total protein. The position of unprocessed (u) and processed (p) large subunits are indicated along with molecular weight markers. (B) Immunodetection of the small subunit HydA_HIS_. Proteins from crude cell extracts were resolved by SDS/PAGE using 12% w/v acrylamide in Tris‐glycine buffer system. Immunoblots were revealed with antisera against the His tag to detect HydA_HIS_. Each lane was loaded with 120 μg of total protein. The position of unprocessed (u) and processed (p) small subunits are indicated along with molecular weight markers. (C) Rocket immunoelectrophoresis of periplasmic fractions. Periplasmic protein samples were prepared by sucrose/lysozyme/EDTA treatment of cells and 2 μg of protein was electrophoresed through 1% (w/v) agarose containing 3 μL of an anti‐Hyd‐5 serum. Plates were then incubated at 37 °C under a H_2_ atmosphere with benzyl viologen and tetrazolium red.

To allow facile identification of the large subunit, new strains were engineered here that produce a HA‐tagged version of the large subunit. Whole‐cell hydrogenase activity was not affected by the addition of the epitope tag (Table 2) and rocket immunoelectrophoresis showed similar levels of active Hyd‐5 were present in the periplasms of the parent strain (LB03) and the modified strain (MAS05; Fig. 1C). Western immunoblotting also clearly identified HydB_HA_ (Fig. 1A) and the processed form of the small subunit HydA_HIS_ (Fig. 1B).

**Table 2:** Effect on hydrogenase activity of the incorporation of a HA‐tag at the N terminus of HydB encoded at its chromosomal locus

Strains	Genotype	Hydrogenase activity
LT2a	wt	23 ± 6
LB03	P_T5_, *hydA* ^ΔTM‐His^	100 ± 12
MAS01	Δ*hypD*	No activity
MAS05	*hydB*_*HA*_	93 ± 2

The absolute values (100%) of hydrogenase activity of anaerobic cells for LB03 strain were 153 ± 20 nmol H_2_ oxidized·min^−1^·mg protein^−1^. Values are means of at least three independent assays ± SE.

A further set of deletion alleles were introduced into the MAS05 strain (Table S1). First, a Δ*hypD* allele was introduced, thus completely blocking [NiFe] cofactor biosynthesis 11. This led to unprocessed large and small subunits (Fig. 1A,B) and the complete loss of periplasmic Hyd‐5 activity (Fig. 1C).

Next, single Δ*hydD* and Δ*hyaD* alleles were introduced into the *hydB*_HA_ background (Table S1). The removal of HyaD had a minor effect on the assembly and activity of Hyd‐5 (Fig. 1), while deletion of *hydD* resulted in a reduction in the cellular levels of Hyd‐5, as observed by rocket immunoelectrophoresis (Fig. 1C) and western immunoblotting (Fig. 1A,B), but the large and small subunits were processed as normal (Fig. 1).

Next, a Δ*hyaD* Δ*hydD* double mutant was constructed (Table S1). This strain was found to be completely devoid of periplasmic Hyd‐5 activity (Fig. 1C) and both the large subunit (Fig. 1A) and small subunit (Fig. 1B) remained in unprocessed forms. Hyd‐5 assembly and activity could be recovered by addition of plasmid‐borne *hyaD* or *hydD* to the double mutant (Fig. 1C), but it is notable that HyaD was unable to restore Hyd‐5 to original levels (Fig. 1C).

### Genetic evidence for a cofactor‐dependent interaction between HydB and its maturation protease

**Figure 2:**
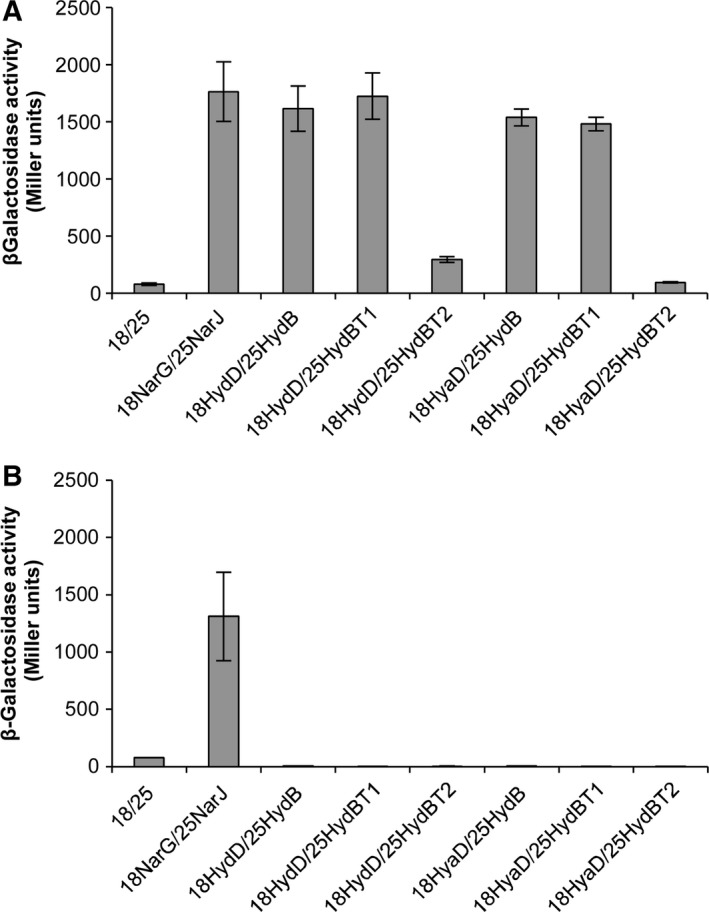
Genetic analysis of HydD‐HydB and HyaD‐HydB interactions using a bacterial two‐hybrid assay. Interaction of HydD or HyaD with HydB and two truncated forms of HydB: HydB_T1_, containing the mature form of HydB; or HydB_T2_, containing a deletion of DNA encoding the C‐terminal 65 amino acid residues of HydB, was quantified by β‐galactosidase activity assays in extracts obtained from anaerobic cultures of (A) *Escherichia coli* MAE01 (Δ*cyaA*) or (B) MAE02 (Δ*cyaA* Δ*hypF*) strains. *E*. *coli* reporter strains transformed with the empty vectors, pUT18 (18) and pT25 (25), or pUT18‐NarG_ss_ (18NarG) and pT25‐NarJ (25NarJ), were included as negative or positive controls, respectively. Values, expressed as Miller units, are the average of three independent assays ± SE.

The relationship between the Hyd‐5 large subunit, HydB, and its maturation proteases, HydD and HyaD, was further studied using a bacterial two‐hybrid assay based on reconstitution of *Bordetella pertussis* adenylate cyclase activity in an *E. coli cyaA* mutant 21. HydD and HyaD were genetically fused to the N terminus of the T18 domain, and the full‐length HydB protein was genetically fused to the C terminus of the T25 domain, of the adenylate cyclase (Table S2). A Δ*cyaA E. coli* reporter strain (MAE01) was generated (Table S1) before being cotransformed with pT25‐HydB and either pUT18‐HydD or pUT18‐HyaD. The transformants were then grown anaerobically in rich medium and subsequent quantification of cellular β‐galactosidase levels showed that the negative control sample (empty vectors) displayed only a basal level of activity (Fig. 2A), while the strain producing the T25‐HydB fusion together with the HydD‐T18 fusion showed high levels of activity (Fig. 2A), indicative of an interaction between HydB and HydD. The observed level of β‐galactosidase induction was similar to that observed for the NarG‐NarJ positive control interaction (Fig. 2A) 32, 33. Next, the T25‐HydB fusion was coproduced with the HyaD‐T18 fusion anaerobically in the *E. coli* reporter strain (Fig. 2A). In this case, an interaction between HydB and HyaD was also clearly detected (Fig. 2A).

The role of the cleavable C‐terminal assembly peptide on the Hyd‐5 catalytic subunit was explored by preparing aT25‐HydB fusion that lacked the final 15 amino acids (termed HydB_T1_). Interestingly, coexpression of truncated T25‐HydB_T1_ with either HydD‐T18 or HyaD‐T18 generated high levels of β‐galactosidase activity, indicating the protein–protein interactions remained intact (Fig. 2A). Next, the HydB large subunit was truncated still further by removal of the final 65 amino acids of HydB, including the DPCXXCXXH motif (HydB_T2_). In this case, coexpression of T25‐HydB_T2_ with either HydD‐T18 or HyaD‐T18 lead to β‐galactosidase activity being greatly reduced closer to that observed for the negative control (Fig. 2A).

To investigate the role of the [NiFe] cofactor in the protein–protein interactions, the *E. coli* Δ*cyaA* reporter strain was further modified by incorporation of a Δ*hypF* allele to give strain MAE02. Next, the Δ*cyaA* Δ*hypF* reporter strain (MAE02) was transformed with the plasmids encoding fusions to the Hyd‐5 components and the results of these experiments revealed that the levels of interactions detected were reduced to basal levels in all cases (Fig. 2B). Note, however, that the values obtained for the NarG‐NarJ positive control, which is a biological system that does not utilize the hydrogenase [NiFe] cofactor, remained strong upon removal of *hypF* (Fig. 2B).

### Biochemical evidence for a stable interaction between HydB and its maturation proteases

**Figure 3:**
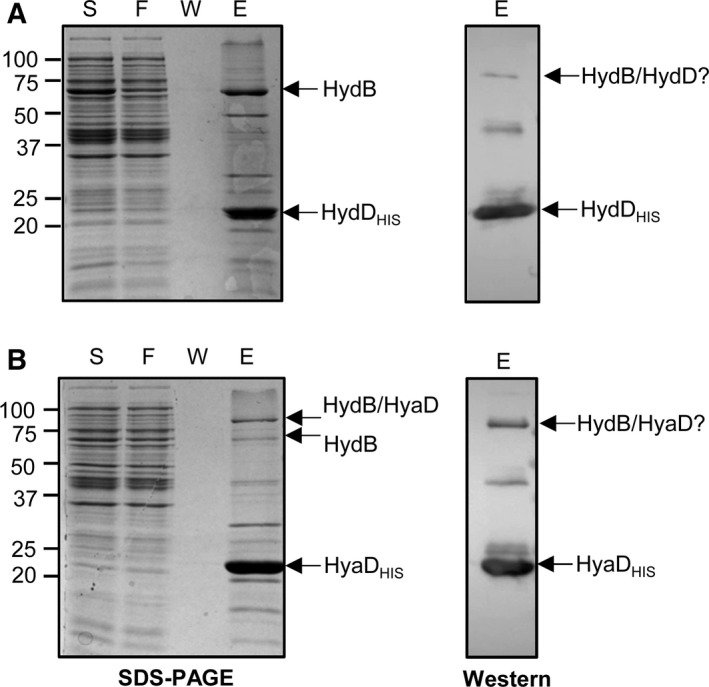
Biochemical analysis of the HydD‐HydB and HyaD‐HydB interactions by copurification experiments. Extracts from anaerobic cultures of *Escherichia coli* FTD147 (Δ*hyaB*, Δ*hybC*, Δ*hycE*) harboring (A) pQE80‐HydB‐HydD_HIS_ or (B) pQE80‐HydB‐HyaD_HIS_ derivative plasmids were applied to a 5 mL HisTrap‐HP IMAC column and eluted fractions were pooled and concentrated. Proteins were resolved in 12% acrylamide SDS/PAGE gels and stained with Instant Blue (left panels) or immunoblotted using an antiserum against His tag (right panels). The arrows indicate the band corresponding to HydB identified by mass spectrometry or HydD_HIS_ and HyaD_HIS_ identified by western immunoblot analysis. Numbers on the left margins of the panels indicate the position of the molecular weight standards (kDa). S, soluble fraction; FT, flow through; W, wash; E, eluate fraction.

The *E. coli* FTD147 strain (Table S1), that lacks the genes for the large subunits of Hyd‐1, Hyd‐2, and Hyd‐3, was transformed with pQE80‐HydB‐HydD_HIS_ and grown under anaerobic induction conditions to allow co‐overproduction of HydB and HydD_HIS_. A soluble protein fraction was prepared and analyzed by immobilized metal ion affinity chromatography (IMAC; Fig. 3A). The HydD_HIS_ protein was found to copurify with a protein of ~ 60 kDa (Fig. 3A). Tryptic peptide mass fingerprinting identified this protein as the *S. enterica* HydB polypeptide (score 43 810) with 96% overall sequence coverage (Fig. S1A). The mass spectrometry demonstrated that the C‐terminal assembly peptide was still present on HyaB (Fig. S1).

A similar approach was taken for HydB and HyaD_HIS_ (Fig. 3B). In this case, only a weakly staining protein was present at ~ 60 kDa (Fig. 3B), but nevertheless tryptic peptide mass analysis identified *S. enterica* HydB (score 17 697) with 96% overall sequence coverage (Fig. S1B). Interestingly, a strongly staining band at ~ 90 kDa was present as copurifying with HyaD_HIS_ and it was observed to cross‐react with the anti‐His antibody used (Fig. 3B). Tryptic peptide analysis of this species identified *S. enterica* HydB, (score 5146 with 65% overall sequence coverage; Fig. S2), together with HyaD (score 1022 with 66% overall sequence coverage; Fig. S2). One interpretation of these data is that the strongly staining band at ~ 90 kDa represents a stable complex between the large subunit and the maturation protease. It could be that the ~ 90 kDa band is a covalent complex between HydD and HyaD, especially as HyaD has three nonconserved cysteines and the samples were not boiled before SDS/PAGE due to the presence of imidazole 34. However, note that some protein oligomers remain stable in SDS/PAGE without covalent bonds 35.

## Discussion

### Cross‐talk between accessory proteins during [NiFe]‐hydrogenase biosynthesis

In organisms that contain several [NiFe]‐hydrogenases, genetic analysis usually points to each large subunit gene being coexpressed with a gene coding for a specific maturation protease 11. In this work, the organization of the operon encoding Hyd‐5 suggested that *hydD* was the most likely candidate as a maturation protease 7. However, data presented here show that, when Hyd‐5 is overproduced under anaerobic conditions, the role of HydD can be partially compensated for by HyaD, which is encoded within the operon for Hyd‐1 (Fig. 1 and Table 1). This was rather unexpected since studies of *E. coli* Hyd‐3 processing demonstrated that protease gene deletions (Δ*hycI* or Δ*hycHI*) were sufficient to block Hyd‐3 biosynthesis 15 but had no effect on the assembly of other hydrogenases in that organism 36. Likewise, the *hoxM* gene product is the essential maturation protease for the Hyd‐5‐like membrane‐bound‐hydrogenase from *Ralstonia eutropha* 37, but the coexpressed soluble hydrogenase requires the action of an alternative gene, *hoxW*, for its maturation 38. It is worth considering, however, that although it has been shown here that *S. enterica* HydD and HyaD are at least partly interchangeable, it is unlikely that the two proteins would be coexpressed under physiological conditions 5. Thus, it remains of critical importance that each differentially expressed isoenzyme is coproduced with its own dedicated maturation protease.

### A stable complex requires cofactor biosynthesis

The two‐hybrid system used here has proven useful in dissection of metalloenzyme biosynthesis pathways and can detect positive interactions that have been subsequently quantified by biophysical techniques to have dissociation constants in the region of 1–1000 nm 32, 33, 39, 40, 41. In this work, an interaction between HydB and HydD could be readily detected using this system (Fig. 2). This behavior in the two‐hybrid assay was initially surprising and counterintuitive. This is because one interpretation of the available literature would be that a maturation protease would recognize its large subunit substrate only transiently when the [NiFe] cofactor was successfully loaded 19, 42, 43. Cleavage might be expected to follow quickly and thus the interaction between protease and large subunit might be short‐lived, transient and unstable 43. Instead, these data suggest the interaction is prolonged and stable under the conditions tested (Fig. 2). The two‐hybrid data also indicate that binding is critically dependent on the presence of the [NiFe] cofactor, since no interactions could be detected in a cofactor mutant (Fig. 2B), but surprisingly points to the interaction being prolonged and stable in the presence of cofactor (Fig. 2A). It is possible that either the large subunit or protease fusions are unstable or degraded in the absence of [NiFe] cofactor, and that this leads to the negative results obtained (Fig. 2B).

Furthermore, the two‐hybrid assay clearly shows that the C‐terminal extension on the large subunit cannot be the key docking position for the maturation proteases (Fig. 2). This observation helps to partly explain recent work on Hyd‐2 assembly in *E. coli* 44. In that work, the C‐terminal extension from the Hyd‐1 large subunit was transposed on to the Hyd‐2 large subunit and processing of the chimera was reported to remain HybD dependent 44. However, conversely, a similar experiment where the Hyd‐2 large subunit extension was transposed onto the Hyd‐3 catalytic domain resulted in a chimera that could not be processed by any maturation protease tested 15.

Interestingly, while HydD and HyaD retained the ability to bind to the mature form of HydB (i.e. that lacking the C‐terminal extension) this interaction was also found to be dependent on an intact [NiFe] cofactor biosynthesis pathway, in so far as the bacterial two‐hybrid data can be interpreted (Fig. 2). This was surprising because so‐truncated large subunit has been previously reported to be completely devoid of the [NiFe] cofactor 42. However, note that in this early work the hydrogenase large subunit in question was subjected to a partial purification procedure that may have led to loss of any loosely bound nickel cofactor that may have been present 42.

Co‐overproduction of the Hyd‐5 large subunit HydB with HydD_HIS_ in an *E. coli* host strain resulted in the isolation of a stable complex between the two proteins (Fig. 3). These data corroborate the two‐hybrid experiments (Fig. 2). Moreover, in this case, the proteolytic event was shown not to have taken place since the C‐terminal peptide extension could be clearly detected by mass spectrometry (Fig. S1A). The ability to isolate such a stable protease‐substrate complex is a fascinating one. A transient hydrogenase‐maturation protease complex was noticed *in vitro* during a metal competition assay 43; however, in most other cases reported, an inactive variant of a protease would have to be prepared in order to obtain a complex such as this. The observation that maturation proteases stably bind to, and do not quickly and transiently proteolytically process their target under these test conditions, adds an unexpected new detail to the hydrogenase biosynthetic pathway.

## Author contributions

MA and FS conceived and coordinated the study and wrote the paper. MA constructed the majority of bacterial strains and plasmids. GB provided technical assistance and constructed a minority of bacterial strains and plasmids. MA and FS designed the experiments and analysed data. MA performed all of the experiments and prepared figures and tables for publication. FS supervised the work.

## Supporting information


**Fig. S1.** Identification of HydB copurified with HydD_HIS_ or HyaD_HIS_.
**Fig. S2.** Identification of a HydB‐HyaD_HIS_ complex.
**Fig. S3.** Sequence identity shared between *Salmonella enterica* maturation proteases.
**Table S1.** Bacterial strains constructed and studied in this work.
**Table S2.** Plasmids used in this work.
**Table S3.** Oligonucleotides used in this work.

## References

[1] Maier RJ , Olczak A , Maier S , Soni S and Gunn J (2004) Respiratory hydrogen use by *Salmonella enterica* serovar Typhimurium is essential for virulence. Infect Immun 72, 6294–6299. 15501756 10.1128/IAI.72.11.6294-6299.2004 PMC523013

[2] Maier L , Barthel M , Stecher B , Maier RJ , Gunn JS and Hardt WD (2014) *Salmonella* Typhimurium strain ATCC14028 requires H2‐hydrogenases for growth in the gut, but not at systemic sites. PLoS One 9, e110187. 25303479 10.1371/journal.pone.0110187 PMC4193879

[3] Lamichhane‐Khadka R , Benoit SL , Miller‐Parks EF and Maier RJ (2015) Host hydrogen rather than that produced by the pathogen is important for *Salmonella enterica* serovar Typhimurium virulence. Infect Immun 83, 311–316. 25368112 10.1128/IAI.02611-14 PMC4288883

[4] Sawers RG , Jamieson DJ , Higgins CF and Boxer DH (1986) Characterization and physiological roles of membrane‐bound hydrogenase isoenzymes from *Salmonella* Typhimurium. J Bacteriol 168, 398–404. 3531177 10.1128/jb.168.1.398-404.1986 PMC213464

[5] Zbell AL , Benoit SL and Maier RJ (2007) Differential expression of NiFe uptake‐type hydrogenase genes in *Salmonella enterica* serovar Typhimurium. Microbiology 153, 3508–3516. 17906148 10.1099/mic.0.2007/009027-0

[6] Lukey MJ , Parkin A , Roessler MM , Murphy BJ , Harmer J , Palmer T , Sargent F and Armstrong FA (2010) How *Escherichia coli* is equipped to oxidize hydrogen under different redox conditions. J Biol Chem 285, 3928–3938. 19917611 10.1074/jbc.M109.067751 PMC2823535

[7] Parkin A , Bowman L , Roessler MM , Davies RA , Palmer T , Armstrong FA and Sargent F (2012) How *Salmonella* oxidises H(2) under aerobic conditions. FEBS Lett 586, 536–544. 21827758 10.1016/j.febslet.2011.07.044

[8] Bowman L , Flanagan L , Fyfe PK , Parkin A , Hunter WN and Sargent F (2014) How the structure of the large subunit controls function in an oxygen‐tolerant [NiFe]‐hydrogenase. Biochem J 458, 449–458. 24428762 10.1042/BJ20131520 PMC3940037

[9] Zbell AL , Maier SE and Maier RJ (2008) *Salmonella enterica* serovar Typhimurium NiFe uptake‐type hydrogenases are differentially expressed in vivo. Infect Immun 76, 4445–4454. 18625729 10.1128/IAI.00741-08 PMC2546827

[10] Fontecilla‐Camps JC , Volbeda A , Cavazza C and Nicolet Y (2007) Structure/function relationships of [NiFe]‐ and [FeFe]‐hydrogenases. Chem Rev 107, 4273–4303. 17850165 10.1021/cr050195z

[11] Böck A , King PW , Blokesch M and Posewitz MC (2006) Maturation of hydrogenases. Adv Microb Physiol 51, 1–71. 17091562 10.1016/s0065-2911(06)51001-x

[12] Rossmann R , Maier T , Lottspeich F and Böck A (1995) Characterisation of a protease from *Escherichia coli* involved in hydrogenase maturation. Eur J Biochem 227, 545–550. 7851435 10.1111/j.1432-1033.1995.tb20422.x

[13] Rossmann R , Sauter M , Lottspeich F and Böck A (1994) Maturation of the large subunit (HYCE) of *Escherichia coli* hydrogenase 3 requires nickel incorporation followed by C‐terminal processing at Arg537. Eur J Biochem 220, 377–384. 8125094 10.1111/j.1432-1033.1994.tb18634.x

[14] Magalon A and Böck A (2000) Dissection of the maturation reactions of the [NiFe] hydrogenase 3 from *Escherichia coli* taking place after nickel incorporation. FEBS Lett 473, 254–258. 10812085 10.1016/s0014-5793(00)01542-8

[15] Theodoratou E , Paschos A , Mintz‐Weber S and Böck A (2000) Analysis of the cleavage site specificity of the endopeptidase involved in the maturation of the large subunit of hydrogenase 3 from *Escherichia coli* . Arch Microbiol 173, 110–116. 10795682 10.1007/s002039900116

[16] Fritsche E , Paschos A , Beisel HG , Böck A and Huber R (1999) Crystal structure of the hydrogenase maturating endopeptidase HYBD from *Escherichia coli* . J Mol Biol 288, 989–998. 10331925 10.1006/jmbi.1999.2719

[17] Kumarevel T , Tanaka T , Bessho Y , Shinkai A and Yokoyama S (2009) Crystal structure of hydrogenase maturating endopeptidase HycI from *Escherichia coli* . Biochem Biophys Res Commun 389, 310–314. 19720045 10.1016/j.bbrc.2009.08.135

[18] Yang F , Hu W , Xu H , Li C , Xia B and Jin C (2007) Solution structure and backbone dynamics of an endopeptidase HycI from *Escherichia coli*: implications for mechanism of the [NiFe] hydrogenase maturation. J Biol Chem 282, 3856–3863. 17150961 10.1074/jbc.M609263200

[19] Theodoratou E , Paschos A , Magalon A , Fritsche E , Huber R and Böck A (2000) Nickel serves as a substrate recognition motif for the endopeptidase involved in hydrogenase maturation. Eur J Biochem 267, 1995–1999. 10727938 10.1046/j.1432-1327.2000.01202.x

[20] Guzman LM , Belin D , Carson MJ and Beckwith J (1995) Tight regulation, modulation, and high‐level expression by vectors containing the arabinose PBAD promoter. J Bacteriol 177, 4121–4130. 7608087 10.1128/jb.177.14.4121-4130.1995 PMC177145

[21] Karimova G , Pidoux J , Ullmann A and Ladant D (1998) A bacterial two‐hybrid system based on a reconstituted signal transduction pathway. Proc Natl Acad Sci USA 95, 5752–5756. 9576956 10.1073/pnas.95.10.5752 PMC20451

[22] Hamilton CM , Aldea M , Washburn BK , Babitzke P and Kushner SR (1989) New method for generating deletions and gene replacements in *Escherichia coli* . J Bacteriol 171, 4617–4622. 2548993 10.1128/jb.171.9.4617-4622.1989 PMC210259

[23] Gust B , Challis GL , Fowler K , Kieser T and Chater KF (2003) PCR‐targeted *Streptomyces* gene replacement identifies a protein domain needed for biosynthesis of the sesquiterpene soil odor geosmin. Proc Natl Acad Sci USA 100, 1541–1546. 12563033 10.1073/pnas.0337542100 PMC149868

[24] Baba T , Ara T , Hasegawa M , Takai Y , Okumura Y , Baba M , Datsenko KA , Tomita M , Wanner BL and Mori H (2006) Construction of *Escherichia coli* K‐12 in‐frame, single‐gene knockout mutants: the Keio collection. Mol Syst Biol 2, 2006.0008. 10.1038/msb4100050 PMC1681482 16738554

[25] Ballantine SP and Boxer DH (1985) Nickel‐containing hydrogenase isoenzymes from anaerobically grown *Escherichia coli* K‐12. J Bacteriol 163, 454–459. 3894325 10.1128/jb.163.2.454-459.1985 PMC219143

[26] Lowry OH , Rosebrough NJ , Farr AL and Randall RJ (1951) Protein measurement with the Folin phenol reagent. J Biol Chem 193, 265–275. 14907713

[27] Ladant D and Karimova G (2000) Genetic systems for analyzing protein‐protein interactions in bacteria. Res Microbiol 151, 711–720. 11130861 10.1016/s0923-2508(00)01136-0

[28] Miller JH (1972) Experiments in Molecular Genetics. Cold Spring Harbor Laboratory, Cold Spring Harbor, NY.

[29] Laemmli UK (1970) Cleavage of structural proteins during the assembly of the head of bacteriophage T4. Nature 227, 680–685. 5432063 10.1038/227680a0

[30] Kneuper H , Cao ZP , Twomey KB , Zoltner M , Jäger F , Cargill JS , Chalmers J , van der Kooi‐Pol MM , van Dijl JM , Ryan RP *et al* (2014) Heterogeneity in *ess* transcriptional organization and variable contribution of the Ess/type VII protein secretion system to virulence across closely related *Staphylocccus aureus* strains. Mol Microbiol 93, 928–943. 25040609 10.1111/mmi.12707 PMC4285178

[31] Towbin H , Staehelin T and Gordon J (1979) Electrophoretic transfer of proteins from polyacrylamide gels to nitrocellulose sheets: procedure and some applications. Proc Natl Acad Sci USA 76, 4350–4354. 388439 10.1073/pnas.76.9.4350 PMC411572

[32] Zakian S , Lafitte D , Vergnes A , Pimentel C , Sebban‐Kreuzer C , Toci R , Claude JB , Guerlesquin F and Magalon A (2010) Basis of recognition between the NarJ chaperone and the N‐terminus of the NarG subunit from *Escherichia coli* nitrate reductase. FEBS J 277, 1886–1895. 20236317 10.1111/j.1742-4658.2010.07611.x

[33] Ize B , Coulthurst SJ , Hatzixanthis K , Caldelari I , Buchanan G , Barclay EC , Richardson DJ , Palmer T and Sargent F (2009) Remnant signal peptides on non‐exported enzymes: implications for the evolution of prokaryotic respiratory chains. Microbiology 155, 3992–4004. 19778964 10.1099/mic.0.033647-0

[34] Bornhorst JA and Falke JJ (2000) Purification of proteins using polyhistidine affinity tags. Methods Enzymol 326, 245–254. 11036646 10.1016/s0076-6879(00)26058-8 PMC2909483

[35] Chalut C , Remy MH and Masson JM (1999) Disulfide bridges are not involved in penicillin‐binding protein 1b dimerization in *Escherichia coli* . J Bacteriol 181, 2970–2972. 10217796 10.1128/jb.181.9.2970-2972.1999 PMC93747

[36] Sauter M , Bohm R and Böck A (1992) Mutational analysis of the operon (*hyc*) determining hydrogenase 3 formation in *Escherichia coli* . Mol Microbiol 6, 1523–1532. 1625581 10.1111/j.1365-2958.1992.tb00873.x

[37] Bernhard M , Schwartz E , Rietdorf J and Friedrich B (1996) The *Alcaligenes eutrophus* membrane‐bound hydrogenase gene locus encodes functions involved in maturation and electron transport coupling. J Bacteriol 178, 4522–4529. 8755880 10.1128/jb.178.15.4522-4529.1996 PMC178219

[38] Thiemermann S , Dernedde J , Bernhard M , Schroeder W , Massanz C and Friedrich B (1996) Carboxyl‐terminal processing of the cytoplasmic NAD‐reducing hydrogenase of *Alcaligenes eutrophus* requires the *hoxW* gene product. J Bacteriol 178, 2368–2374. 8636040 10.1128/jb.178.8.2368-2374.1996 PMC177947

[39] Vergnes A , Gouffi‐Belhabich K , Blasco F , Giordano G and Magalon A (2004) Involvement of the molybdenum cofactor biosynthetic machinery in the maturation of the *Escherichia coli* nitrate reductase A. J Biol Chem 279, 41398–41403. 15247236 10.1074/jbc.M407087200

[40] Buchanan G , Maillard J , Nabuurs SB , Richardson DJ , Palmer T and Sargent F (2008) Features of a twin‐arginine signal peptide required for recognition by a Tat proofreading chaperone. FEBS Lett 582, 3979–3984. 19013157 10.1016/j.febslet.2008.10.049

[41] Grahl S , Maillard J , Spronk CA , Vuister GW and Sargent F (2012) Overlapping transport and chaperone‐binding functions within a bacterial twin‐arginine signal peptide. Mol Microbiol 83, 1254–1267. 22329966 10.1111/j.1365-2958.2012.08005.x PMC3712460

[42] Binder U , Maier T and Böck A (1996) Nickel incorporation into hydrogenase 3 from *Escherichia coli* requires the precursor form of the large subunit. Arch Microbiol 165, 69–72. 8639025 10.1007/s002030050299

[43] Magalon A , Blokesch M , Zehelein E and Böck A (2001) Fidelity of metal insertion into hydrogenases. FEBS Lett 499, 73–76. 11418115 10.1016/s0014-5793(01)02525-x

[44] Thomas C , Muhr E and Sawers RG (2015) Coordination of synthesis and assembly of a modular membrane‐associated [NiFe]‐hydrogenase is determined by cleavage of the C‐terminal peptide. J Bacteriol 197, 2989–2998. 26170410 10.1128/JB.00437-15 PMC4542169

