# Supplementary material for: Identification of a stable complex between a [NiFe]‐hydrogenase catalytic subunit and its maturation protease
Source: FEBS Lett. 2017 Jan 11;591(2):338–47. doi: 10.1002/1873-3468.12540 (PMC5299533; doi:10.1002/1873-3468.12540)
Supplement: Supplementary file 1 — Fig. S1. Identification of HydB copurified with HydDHIS or HyaDHIS. Fig. S2. Identification of a HydB‐HyaDHIS complex. Fig. S3. Sequence identity shared between Salmonella enterica maturation proteases. Table S1. Bacterial strains constructed and studied in this work. Table S2. Plasmids used in this work. Table S3. Oligonucleotides used in this work. [file FEB2-591-338-s001.pdf]

Identification of a stable complex between a [NiFe]-hydrogenase catalytic subunit and its maturation protease.

Marta Albareda[1], Grant Buchanan and Frank Sargent

School of Life Sciences, University of Dundee, Dundee DD1 5EH, Scotland, UK

SUPPLEMENTARY INFORMATION

Supp. Table S1. Bacterial strains constructed and studied in this work

Strain	Relevant genotype	Source or reference
Salmonella enterica serovar Typhimurium		
LT2a	parental strain	Laboratory stock
SFTH01	as LT2a (*hydA*$_{HIS}$)	(1)
SFTH06a	as SFTH01 (P$_{T5}$,*hydA*$_{HIS}$)	(1)
LB03	as SFTH06 (P$_{T5}$,*hydA*$_{HIS\text{-}\Delta TM}$)	(2)
LB03T	as LB03 (Δ*tatABC*::Apra^{R})	(3)
MAS01	as LB03 (Δ*hypD*)	This work
MAS02	as LB03 (Δ*hydD*)	This work
MAS03	as LB03 (Δ*hyaD*)	This work
MAS04	as LB03 (Δ*hyaD*, Δ*hydD*)	This work
MAS05	as LB03 (*hydB*$_{HA}$)	This work
MAS06	as LB03 (Δ*hypD*, *hydB*$_{HA}$)	This work
MAS07	as LB03 (Δ*hydD*, *hydB*$_{HA}$)	This work
MAS08	as LB03 (Δ*hyaD*, *hydB*$_{HA}$)	This work
MAS09	as LB03 (Δ*hyaD*, Δ*hydD*, *hydB*$_{HA}$)	This work
Escherichia coli		
MG1655	*E. coli* K-12: F-, λ-, *ilvG-*, *rfb-50*, *rph-1*	(4)
BW25113	F-, λ-, Δ(*araD-araB*)*567*, Δ*lacZ4787*(::rrnB-3), Δ(*rhaD-rhaB*)*568*, *rph-1*, *hsdR514*	(5)
JW5433	as BW25113, Δ*hypF*::Kan^{R}	(5)
MAE01	as MG1655 (Δ*cyaA*::Apra^{R})	This work
MAE02	as MG1655 (Δ*cyaA*::Apra^{R}, Δ*hypF*::Kan^{R})	This work
MC4100	F^{-}, Δ*lacU169, araD139, rpsL150, relA1, ptsF, rbs, flbB5301*	(6)
FTD147	as MC4100, Δ*hyaB*, Δ*hybC*, Δ*hycE*	(7)

Supp. Table S2. Plasmids used in this work

Plasmids	Description	Source or reference
pBlueScript KS+	High copy cloning vector (Amp^R)	Stratagene
pMAK705	Temperature-sensitive vector (Cml^R)	(8)
pMAK-hypD	pMAK705 derivative containing ~ 500 bp up- and downstream of *hypD* (Δ*hypD* allele)	This work
pMAK-hyaD	pMAK705 carrying Δ*hyaD* allele	This work
pMAK-hydD	pMAK705 carrying Δ*hydD* allele	This work
pBAD24	Arabinose-inducible expression vector (Amp^R)	(9)
pBADHyaD	pBAD24 derivative carrying *hyaD*	This work
pBADHydD	pBAD24 derivative carrying *hydD*	This work
pUT18	Amp^R (ori pMB1) vector for generation of N-terminal T18 fusions.	(10)
pUT18-$NarG_{SS}$	pUT18 derivative containing the 36 codons at the 5' end of *narG* gene	(11)
pUT18-HydD	pUT18 derivative carrying fusion to *hydD*	This work
pUT18-HyaD	pUT18 derivative carrying fusion to *hyaD*	This work
pT25	Cm^R (p15A) vector for generation of C-terminal T25 fusions	(10)
pT25-NarJ	pT25 derivative carrying *narJ* fusion	(11)
pT25-HydB	pT25 derivative carrying *hydB* fusion	
pT25-$HydB_{T1}$	pT25 derivative containing fusion to truncated *hydB* lacking final 15 codons	This work
pT25-$HydB_{T2}$	pT25 derivative containing fusion to truncated *hydB* lacking final 65 codons	This work
pFGM1	pMAK705 derivative containing Δ*cyaA* allele	This work
pIJ773	pBlueScript KS+ derivative containing $Apra^R$ cassette and *oriT* flanked by FRT sites	(12)
pFGM2	As pFGM1 with $Apra^R$ cassette giving Δ*cyaA*::$Apra^R$ allele	This work
pQE80L	Protein over production vector (AmpR)	Qiagen
pQE80-HydB	pQE80-L derivative carrying *hydB* gene	This work
pQE80-HydB-$HyaD_{HIS}$	pQE80-HydB derivative carrying $hyaD_{HIS}$	This work
pQE80-HydB-$HydD_{HIS}$	pQE80-HydB derivative carrying $hydD_{HIS}$	This work

Supplementary Table S3. Oligonucleotides used in this work

Primer	Sequence (5´-3´)	Use
1hypD_BamHI_FW	GCGGGATCCATAAAGTCGCAGTGCTTTCC	*hypD* deletion; amplification of DNA upstream of *hypD* gen
2hypD_EcoRI_RV	GCGGAATTCCGTCAACAAAACGCATTATC	
3hypD_EcoRI_FW-2	GCGGAATTCAGGAGTGTGAAGTTTGAAC	*hypD* deletion; amplification of DNA downstream of *hypD* gen
4hypD_HindIII_RV	GCGAAGCTTCGCCAACGCTCAGCGTTTGC	
1hydD_BamHI_FW	GCGGGATCCCGACTGATCCATTTTACCAC	*hydD* deletion; amplification of DNA upstream of *hydD* gen
2hydD_EcoRI_RV	GCGGAATTCGATTGTTACTTCTGCCATTG	
3hydD_EcoRI_FW	GCGGAATTCCAGAGAGAGAAATCATAATG	*hydD* deletion; amplification of DNA downstream of *hydD* gen
4hydD_HindIII_RV	GCGAAGCTTCAAATGCCGCGGTATAAGAC	
1hyaD_BamHI_FW	GCGGGATCCGTTACTGCTGGGGCGAATTT	*hyaD* deletion; amplification of DNA upstream of *hyaD* gen
2hyaD_EcoRI_RV	GCGGAATTCTACGCGTTGCGCATTCATGA	
3hyaD_EcoRI_FW	GCGGAATTCAAAAGAAGTGGCGAATGACAC	*hyaD* deletion; amplification of DNA downstream of *hyaD* gen
4hyaD_HindIII_RV	GCGAAGCTTGGTAAGGGATTCATCGAGAA	
pBADHyaD_EcoRI_FW	GCGGAATTCATGAATGCGCAACGCGTAGT	Cloning HyaD in pBAD24 vector
pBADHyaD_SalI_RV	GCGGTCGACTCATTCGCCACTTCTTTTGC	
pBADHydD_EcoRI_FW	GCGGAATTCATGGCAGAAGTAACAATCTT	Cloning HydD in pBAD24 vector
pBADHydD_SalI_RV	GCGGTCGACTTATGATTTCTCTCTCTGTA	
1HA tag HydB _SacI_FW	GCGGAGCTCAACCCAACTAAAGCCACGCC	Incorporation of a HA tag at the N-terminus of HydB
2HA tag HydB _RV	AGCGTAATCTGGAACATCGTATGGGTACATAATT ATTTTTTCTCTTCCGGAGCA	
3HA tag HydB _FW	ATGTACCCATACGATGTTCCAGATTACGCTGCAT ATCCTTATCAGACTCAGGGTTTT	

4HA tag<br>HydB_XbaI_RV	GCGTCTAGAAGGGCCAAGTTGACCGGACTC	
T18HyaD_BamHI_FW<br>T18HyaD_EcoRI_RV	GCGGGATCCATGAATGCGCAACGCGTA<br>GCGGAATTCGATTCGCCACTTCTTTTGCC	Cloning HyaD in pUT18 vector
T18HydD_BamHI_FW<br>T18HydD_EcoRI_RV	GCGGGATCCATGGCAGAAGTAACAATCTT<br>GCGGAATTCGATGATTTCTCTCTCTGTA	Cloning HydD in pUT18 vector
T25HydBPstIfor<br>T25HydBBamHIrev	GCGCGAATTCCTGCAGCCGCATATCCTTATCAGACTCAGG<br>GCGCGGATCCTCATCGTACCTGAACCCTGACCAGC	Cloning HydB in pT25 vector
T25HydB_BamHI_FW<br>T25HydBtrun_SmaI_RV	GCGGGATCCCGCATATCCTTATCAGACTCA<br>GCGCCCGGGTCAATGGGTGGAGCAGGCCAGGCAGGGATC	Cloning $HydB_{T1}$ in pT25 vector
T25HydB_BamHI_FW<br>T25HydBtrun_KpnI_RV	GCGGGATCCCGCATATCCTTATCAGACTCA<br>GCGGGTACCTCAATGGGTGGAGCAGGCCAGGCAGGGATC	Cloning $HydB_{T2}$ in pT25 vector
DelcyaforEcoRIKpn-I<br>Del-cyaA2	GCGCGAATTCGGTACCAGTTCAACGACCAG<br>GCGCTCTAGAGAGGTACAAGACGTATCGC	*cyaA* deletion; amplification of DNA upstream of *cyaA gen*
Del-cyaA3<br>Del-cyaA4	GCGCTCTAGATTTTCGTGATGAACGTGCCGG<br>GCGCAAGCTTACAAACTTTCGCTACACAG	*cyaA* deletion; amplification of DNA upstream of *cyaA gen*
ApraforSpeI-5’<br>AprarevSpeI-3’	GCGCACTAGTATTCCGGGGATCCGTCGACC<br>GCGCACTAGTTGTAGGCTGGAGCTGCTTC	Amplification of apramycin cassette
HydB-80-EcoRI-FW<br>HydB-80-SphI-Rv	GCGCGAATTCCACAGAGGAACAGGTATGGCATATCCTTATCAGACTCAGGGT<br>GCGCGCATGCTTATTATCGTACCTGAACCCTGACC	Cloning HydB in pQE80 vector
HyaD-80-PstI-FW<br>HyaD-80-HindIII-RV	GCGCCTGCAGCACAGAGGAACAGGTATGAATGCGCAACGCGTAGTGGTGA<br>GCGCAAGCTTTTATTAGTGATGGTGATGGTGATGTTCGCCACTTCTTTTGCCCTCCAGA	Cloning HyaD in pQE80 vector

HydD-80-PstI-FW	GCGCCTGCAGCACAGAGGAACAGGTATGGCAG AAGTAACAATCTTAGGGCT	Cloning HydD in pQE80 vector
HydD-80-HindIII-RV	GCGCAAGCTTTTATTAGTGATGGTGATGGTGAT GTGATTTCTCTCTCTGTAGCACCCGA	

SUPPLEMENTARY FIGURES

Supp. Figure S1. Identification of HydB co-purified with $HydD_{HIS}$ or $HyaD_{HIS}$. HydB was identified by Tryptic peptide mass fingerprinting in eluted fractions obtained from HisTrap-HP immobilized metal ion affinity chromatography (IMAC) column loaded with extracts from *E. coli* FTD147 derivative strains carrying pQE80HydB-$HydD_{HIS}$ (**A**) or pQE80HydB-$HyaD_{HIS}$ (**B**). HydB was identified with a 96 % sequence coverage (**A**, score 43810 and **B**, score 17697) and showed that the C-terminal peptide was present.

A.

Matched peptides shown in ***bold red***.

```
  1 MAYPYQTQGF TLDNSGRRIV VDPVTRIEGH MRCEVNIDSN NVITNAVSTG
 51 TMWRGLEVIL KGRDPRDAWA FVERICGVCT GTHALTSIRA VENALGIAIP
101 DNANCIRNMM QATLHVHDHL VHFYHLHALD WVDVVAALKA DPHQTSAIAQ
151 SLSAWPLSSP GYFRDLQNRL KRFIESGQLG PFRNGYWGHP AMKLPPEANL
201 LAVAHYLEAL DFQKEIVKIH TVFGGKNPHP NWLVGGVPCA INLDETGAVG
251 AVNMERLNLV SSIIQKARQF CEQVYLPDVL LIASYYKDWA KIGGGLSSMN
301 LLAYGEFPDN PNDYSASNLL LPRGAIINGR FDEIHPVDLT APDEIQEFVT
351 HSWYTYGNGN NDKGLHPWDG LTEPQLVMGE HYKGTKTFIE QVDESAKYSW
401 IKSPRWKGHA MEVGPLARYL IGYHQNKPEF KEPVDQLLSV LKLPKEALFS
451 TLGRTAARAL ESVWAGNTLQ YFFDRLMRNL KSGDTATANV TLWEPDTWPT
501 SAKGVGFSEA PRGALGHWIK IANQKIDSYQ CVVPTTWNAG PRDDKGQIGA
551 YEAALMGTKL AVPDQPLEIL RTLHSFDPCL ACSTHVIDNH GGELVRVQVR
```

B.

Matched peptides shown in ***bold red***.

```
  1 MAYPYQTQGF TLDNSGRRIV VDPVTRIEGH MRCEVNIDSN NVITNAVSTG
 51 TMWRGLEVIL KGRDPRDAWA FVERICGVCT GTHALTSIRA VENALGIAIP
101 DNANCIRNMM QATLHVHDHL VHFYHLHALD WVDVVAALKA DPHQTSAIAQ
151 SLSAWPLSSP GYFRDLQNRL KRFIESGQLG PFRNGYWGHP AMKLPPEANL
201 LAVAHYLEAL DFQKEIVKIH TVFGGKNPHP NWLVGGVPCA INLDETGAVG
251 AVNMERLNLV SSIIQKARQF CEQVYLPDVL LIASYYKDWA KIGGGLSSMN
301 LLAYGEFPDN PNDYSASNLL LPRGAIINGR FDEIHPVDLT APDEIQEFVT
351 HSWYTYGNGN NDKGLHPWDG LTEPQLVMGE HYKGTKTFIE QVDESAKYSW
401 IKSPRWKGHA MEVGPLARYL IGYHQNKPEF KEPVDQLLSV LKLPKEALFS
451 TLGRTAARAL ESVWAGNTLQ YFFDRLMRNL KSGDTATANV TLWEPDTWPT
501 SAKGVGFSEA PRGALGHWIK IANQKIDSYQ CVVPTTWNAG PRDDKGQIGA
551 YEAALMGTKL AVPDQPLEIL RTLHSFDPCL ACSTHVIDNH GGELVRVQVR
```

Supp. Figure S2. Identification of a HydB-$HyaD_{HIS}$ complex. HydB was co-purified with $HyaD_{HIS}$ and these proteins were identified by Tryptic peptide mass fingerprinting in eluted fractions obtained from HisTrap-HP immobilized metal ion affinity chromatography (IMAC) column loaded with extracts from *E. coli* FTD147 derivative strains carrying pQE80HydB-$HyaD_{HIS}$. HydB (**A**) was identified with a 65% sequence coverage (score 5146) and HyaD (**B**) with a 66% sequence coverage (score 1022).

A.

Matched peptides shown in ***bold red***.

1 M**AYPYQTQGF** **TLDNSGRRIV** **VDPVTRIEGH** **MRCEVNIDSN** **NVITNAVSTG**

51 **TMWRGLEVIL** **K**GRDPR**DAWA** **FVERICGVCT** **GTHALTSIRA** **VENALGIAIP**

101 **DNANCIR**NMM QATLHVHDHL VHFYHLHALD WVDVVAALK**A** **DPHQTSAIAQ**

151 **SLSAWPLSSP** **GYFR**DLQNRL K**RFIESGQLG** **PFRNGYWGHP** **AMK**LPPEANL

201 LAVAHYLEAL DFQKEIVK**IH** **TVFGGK**NPHP NWLVGGVPCA INLDETGAVG

251 AVNMER**LNLV** **SSIIQKAR**QF CEQVYLPDVL LIASYYKDWA KIGGGLSSMN

301 LLAYGEFPDN PNDYSASNLL LPR**GAIINGR** FDEIHPVDLT APDEIQEFVT

351 HSWYTYGNGN NDK**GLHPWDG** **LTEPQLVMGE** **HYK**GTK**TFIE** **QVDESAKYSW**

401 **IK**SPR**WKGHA** **MEVGPLARYL** **IGYHQNKPEF** **KEPVDQLLSV** **LKLPKEALFS**

451 **TLGRTAARAL** **ESVWAGNTLQ** **YFFDR**LM**RNL** **KSGDTATANV** **TLWEPDTWPT**

501 **SAKGVGFSEA** **PRGALGHWIK** **IANQKIDSYQ** **CVVPTTWNAG** **PRDDKGQIGA**

551 **YEAALMGTKL** **AVPDQPLEIL** **RTLHSFDPCL** **ACSTHVIDNH** **GGELVR**VQVR

B.

Matched peptides shown in ***bold red***.

1 MNAQR**VVVMG** **LGNLLWADEG** **FGIR**VAERLY ARYHWPEEVE IVDGGTQGLN

51 LLGYVEQASH LLLLDAIDYG LAPGSLR**TYA** **GEKIPAYLSA** **KKMSLHQNSF**

101 **SEVLALADIR** **GHLPCHIALV** **GLQPALLDDY** **GGSLSEIARS** **QLPAAEQAAL**

151 **AQLAAWGIVP** **QANEVAR**CLN YK**CLSMENYE** **GVRIRQYQTR** **LEGK**RSGE

Supp. Figure S3. Sequence identity shared between *S.enterica* maturation proteases.

```
HyaD    1 MNAQRVVVMGLGNLLWADEGFGIRVAERLYARYHWPEEVEIVDGGTQGLNLLGYVEQASH
HydD    1 --MAEVTILGLGNLLWADEGFGVRAAEKLFEQYADNEKVDVVDGGTQGLALLPWLQQTEK
HybD    1 ---MRIILVLGVGNILLTDEAIGVRIVEALEQRYTLPDFVEILDGGTAGMELLGDMANRDH

HyaD   61 LLLLDAIDYG-LAPGSLRTYAGEKIPAYLSAKKMSLHQNSFSEVLALADIRGHLPCHIAL
HydD   59 LLIMDAIDFG-MAPGSLAMFRDEQVPAYLTAKKLSLHQTSFSEVLALLQLTGGQLSEIVL
HybD   58 LIIADAIVSKKNAPGTIMVLRDDEVPALFTN-KISPHQLGLADVLSALRFTGEFPKKLTL

HyaD  120 VGLQPALLDDYGGSLSEIARSQLPAAEQAALAQLAAWGIVP-QANEVARCLNYKCLSMEN
HydD  118 IGVQPECLDDYGGSLTPQVKAQLMPAVYLAQEVLAQWGITASSAALPTERLNHYSLCMER
HybD  117 VGVIPQSLEPHIG-LTPTVEAMIEPALEQVLAALRESGVEAIPKETAHV-----------

HyaD  179 YEGVR-IRQYQTRLEGKRSGE----
HydD  178 YEDERPDAQSACRVGDIRVLQREKS
HybD      -------------------------
```

SUPPLEMENTARY REFERENCES

1. Parkin, A., Bowman, L., Roessler, M. M., Davies, R. A., Palmer, T., Armstrong, F. A., and Sargent, F. (2012) How Salmonella oxidises H(2) under aerobic conditions. *FEBS letters* **586**, 536-544
2. Bowman, L., Flanagan, L., Fyfe, P. K., Parkin, A., Hunter, W. N., and Sargent, F. (2014) How the structure of the large subunit controls function in an oxygen-tolerant [NiFe]-hydrogenase. *The Biochemical journal* **458**, 449-458
3. Bowman, L., Balbach, J., Walton, J., Sargent, F., and Parkin, A. (2016) Biosynthesis of Salmonella enterica [NiFe]-hydrogenase-5: probing the roles of system-specific accessory proteins. *J Biol Inorg Chem* **21**, 865-873
4. Blattner, F. R., Plunkett, G., 3rd, Bloch, C. A., Perna, N. T., Burland, V., Riley, M., Collado-Vides, J., Glasner, J. D., Rode, C. K., Mayhew, G. F., Gregor, J., Davis, N. W., Kirkpatrick, H. A., Goeden, M. A., Rose, D. J., Mau, B., and Shao, Y. (1997) The complete genome sequence of Escherichia coli K-12. *Science* **277**, 1453-1462
5. Baba, T., Ara, T., Hasegawa, M., Takai, Y., Okumura, Y., Baba, M., Datsenko, K. A., Tomita, M., Wanner, B. L., and Mori, H. (2006) Construction of Escherichia coli K-12 in-frame, single-gene knockout mutants: the Keio collection. *Mol Syst Biol* **2**, 2006 0008
6. Casadaban, M. J., and Cohen, S. N. (1979) Lactose genes fused to exogenous promoters in one step using a Mu-lac bacteriophage: in vivo probe for transcriptional control sequences. *Proceedings of the National Academy of Sciences of the United States of America* **76**, 4530-4533
7. Redwood, M. D., Mikheenko, I. P., Sargent, F., and Macaskie, L. E. (2008) Dissecting the roles of Escherichia coli hydrogenases in biohydrogen production. *FEMS Microbiol Lett* **278**, 48-55
8. Hamilton, C. M., Aldea, M., Washburn, B. K., Babitzke, P., and Kushner, S. R. (1989) New method for generating deletions and gene replacements in Escherichia coli. *Journal of bacteriology* **171**, 4617-4622
9. Guzman, L. M., Belin, D., Carson, M. J., and Beckwith, J. (1995) Tight regulation, modulation, and high-level expression by vectors containing the arabinose PBAD promoter. *Journal of bacteriology* **177**, 4121-4130
10. Karimova, G., Pidoux, J., Ullmann, A., and Ladant, D. (1998) A bacterial two-hybrid system based on a reconstituted signal transduction pathway. *Proc Natl Acad Sci U S A* **95**, 5752-5756
11. Ize, B., Coulthurst, S. J., Hatzixanthis, K., Caldelari, I., Buchanan, G., Barclay, E. C., Richardson, D. J., Palmer, T., and Sargent, F. (2009) Remnant signal peptides on non-exported enzymes: implications for the evolution of prokaryotic respiratory chains. *Microbiology* **155**, 3992-4004
12. Gust, B., Challis, G. L., Fowler, K., Kieser, T., and Chater, K. F. (2003) PCR-targeted Streptomyces gene replacement identifies a protein domain needed for biosynthesis of the sesquiterpene soil odor geosmin. *Proceedings of the National Academy of Sciences of the United States of America* **100**, 1541-1546
